# The monetary value of human lives lost through Ebola virus disease in the Democratic Republic of Congo in 2019

**DOI:** 10.1186/s12889-019-7542-2

**Published:** 2019-09-03

**Authors:** Joses M. Kirigia, Rose Nabi Deborah Karimi Muthuri, Newton Gitonga Muthuri

**Affiliations:** 1 African Sustainable Development Research Consortium (ASDRC), P.O. Box 6994 00100 GPO, Nairobi, Kenya; 2 Faculty of Health Sciences, University of Pretoria, Pretoria, South Africa; 3 School of Business, United States International University, Nairobi, Kenya

**Keywords:** Ebola virus disease (EVD) deaths, Human capital, Monetary value of life, Years of life lost, Gross domestic product, Democratic Republic of Congo (DRC)

## Abstract

**Background:**

Between 8 May 2018 and 27 May 2019, cumulatively there were 1286 deaths from Ebola Virus Disease (EVD) in the Democratic Republic of Congo (DRC). The objective of this study was to estimate the monetary value of human lives lost through EVD in DRC.

**Methods:**

Human capital approach was applied to monetarily value years of life lost due to premature deaths from EVD. The future losses were discounted to their present values at 3% discount rate. The model was reanalysed using 5 and 10% discount rates. The analysis was done alternately using the average life expectancies for DRC, the world, and the Japanese females to assess the effect on the monetary value of years of life lost (MVYLL).

**Results:**

The 1286 deaths resulted in a total MVYLL of Int$17,761,539 assuming 3% discount rate and DRC life expectancy of 60.5 years. The average monetary value per EVD death was of Int$13,801. About 44.7 and 48.6% of the total MVYLL was borne by children aged below 9 years and adults aged between 15 years and 59 years, respectively.

Re-estimation of the algorithm with average life expectancies of the world (both sexes) and Japanese females, holding discount rate constant at 3%, increased the MVYLL by Int$ 3,667,085 (20.6%) and Int$ 7,508,498 (42.3%), respectively. The application of discount rates of 5 and 10%, holding life expectancy constant at 60.5 years, reduced the MVYLL by Int$ 4,252,785 (− 23.9%) and Int$ 9,658,195 (− 54.4%) respectively.

**Conclusion:**

The EVD outbreak in DRC led to a considerable MVYLL. There is an urgent need for DRC government and development partners to disburse adequate resources to strengthen the national health system and other systems that address social determinants of health to end recurrence of EVD outbreaks.

**Electronic supplementary material:**

The online version of this article (10.1186/s12889-019-7542-2) contains supplementary material, which is available to authorized users.

## Background

The Democratic Republic of the Congo (DRC) is situated in Central Africa [1]. It is divided into 26 administrative provinces and has a population of 97.879 million persons. The country had a total gross domestic product (GDP) of US$ 48.458 billion and a per capita GDP of US$ 495.1 (equivalent to Int$ 791.19) in 2019 [2].

In 2017 DRC had a low human development index (HDI) of 0.457 and inequality-adjusted HDI of 0.319; life expectancy of 60.5 years; and 6.8 mean years of schooling [3]. It had an overall multidimensional poverty index of 0.378. The low standard of living contributes 56.2%, health 25% and education 18.9% to overall multidimensional poverty. Approximately, 57 million of the population live in multidimensional poverty.

The Ministry of Health in DRC notified World Health Organization (WHO) of confirmed cases of ongoing Ebola Virus Disease (EVD) outbreak in the provinces of North Kivu and Ituri on 8 May 2018 [4]. As of 27 May 2019, the cumulative number of EVD cases was 1926. There were 1286 EVD-related deaths between 8 May 2018 and 27 May 2019; of which 91.8 and 8.2% occurred in North-Kivu and Ituri provinces, respectively [5] (See Additional file 1).

The recurrent EVD epidemics could be attributed partially to underinvestment in the national health system and other systems that address social determinants of health [6]. For instance, the current health expenditure (CHE) per capita was US$21 in 2016; which was below the US$74 to US$ 198 total cost per person needed to reach health system targets for the attainment of SDG3 [7]. It was made up of domestic general government health expenditure (GGHE-D) per capita of US$3; domestic private health expenditure (PVT-D) per capita of US$9; and external health expenditure (EXT) per capita of US$9. The DRC out-of-pocket expenditure (OOPS) per capita of US$8 was 88.9% of PVT-D and 38.1% of CHE [8]. According to the WHO World Health Report 2010 [9], when OOPs are above 15–20% of CHE, the incidence of financial catastrophe and impoverishment increases substantially.

The DRC universal health coverage (UHC) service index was 40% in 2015 [6]; implying essential health services coverage gap of 60%. The tracer indicators of the essential health services include reproduction, maternal, newborn and child health (family planning, pregnancy and delivery care, child immunization and treatment); infectious diseases (TB treatment, HIV treatment, malaria prevention, water and sanitation); noncommunicable diseases (treatment of cardiovascular diseases, management of diabetes, cervical cancer screening, tobacco control); and services capacity and access (hospital access, health worker density, essential medicines and health security) [6]. There is a need for sustained evidence-based advocacy for the DRC government, private sector, and external partners to increase their investments in the building of resilient national health system to attain universal coverage of essential health services.

Some of the evidence needed for advocacy is the monetary value of lives lost from a disease such as EVD. A few studies have estimated the economic burden of EVD. For example, Bartsch, Gorham and Lee estimated the cost of an EVD case in Guinea, Liberia and Sierra Leone [10]. Kirigia et al estimated the future productivity losses associated with EVD deaths in Guinea, Liberia, Mali, Nigeria and Sierra Leone [11]. Kastelic et al estimated the socio-economic impacts of Ebola in Sierra Leone [12]. Bowles et al estimated the extent to which economic activity declined and jobs disappeared in Liberia during the outbreak [13]. Thomas, Smith and Ferreira provided an update of the macro-economic impact of Ebola on sub-Saharan Africa in 2015 [14]. There is a dearth of similar economic evidence on the EVD epidemic in DRC.

Thus, the aim of this study is to contribute towards bridging that knowledge gap. The specific objective was to estimate the monetary value of human lives lost from EVD in DRC.

## Methods

### Human capital approach framework

This study employed human capital approach (HCA) to monetarily value potential years of life lost (PYLL) due to premature mortality caused by the EVD in the DRC between 8 May 2018 and 27 May 2019. Culyer [15] defines human capital as the stock of human skills (determined by basic ability, educational attainment and health status) embodied in an individual; which is measured as the present value of expected earnings over a period of time [15].

Death extinguishes expected flow of productive capability in future years. Assuming per capita GDP is a proxy of individual person’s rate of remuneration, the productivity forgone by society because of premature mortality from EVD is the sum of the discounted stream of potential earnings that could have accrued over the potential years of life lost (PYLL) [16]. According to Gardner and Sanborn [17]. PYLL is a measure of the average time a person would have lived had (s)he not died prematurely, i.e. DRC life expectancy at death minus age group’s average age at death.

Thus, potential economic losses to society were estimated using PYLL, a cut-off point at the DRC life expectancy at birth of 60.5 years, 3% discount rate, number of EVD deaths at various age groups, and non-health per capita GDP (per capita GDP minus total current health expenditure). Only the PYLL above the DRC minimum working age for admission to work of 15 years was monetarily valued for age groups 1–4 years, 5–9 years and 10–14 years [18].

The analytical framework formulated below was adapted from the frameworks applied in estimating the indirect costs of maternal deaths in 2010 [19], maternal deaths in 2013 [20], child mortality [21], neglected tropical diseases deaths [22], tuberculosis [23] and non-communicable disease deaths [24] in the WHO African Region, and Ebola Virus Disease in West Africa [11]. As recommended first by Weisbrod [25], and subsequently, by WHO [26] we deducted current health expenditure from gross productivity to obtain net productivity.

The monetary value of potential PYLL lost (*MVYLL*) due to EVD deaths in DRC is the sum of the potential non-health GDP lost among those aged 1–4 (*MVYLL*_1 − 4_), those aged 5–9 (*MVYLL*_5 − 9_), those aged 10–14 (*MVYLL*_10 − 14_), those aged 15–19 (*MVYLL*_15 − 19_), those aged 20–24 (*MVYLL*_20 − 24_), those aged 25–29 (*MVYLL*_25 − 29_), those aged 30–34 (*MVYLL*_30 − 34_), those aged 35–39 (*MVYLL*_35 − 39_), those aged 40–44 (*MVYLL*_40 − 44_), those aged 45–49 (*MVYLL*_45 − 49_), those aged 50–54 (*MVYLL*_50 − 54_), those aged 55–59 (*MVYLL*_55 − 59_), those aged 60–64 (*MVYLL*_60 − 64_), those aged 65–69 (*MVYLL*_65 − 69_), those aged 70–74 (*MVYLL*_70 − 74_), those aged 75–79 (*MVYLL*_75 − 79_), those aged 80–84 (*MVYLL*_80 − 84_), those aged 85–89 (*MVYLL*_85 − 89_), those aged 90–94 (*MVYLL*_90 − 94_), and those aged 95 years and above (*MVYLL*_≥95_).

The MVYLL among persons of a specific age group is the product of the total discounted years of life lost, per capita non-health GDP in purchasing power parity (NHGPC_Int$_) and the total number of EVD deaths (EVDD) for age group [11, 19–24]. The DRC’s discounted total non-health GDP loss attributable to EVD deaths (MVYLL_DRC_) was estimated using the eqs. (1) and (2) below [11] (see Additional file 2 for details).

1 $$ {MVYLL}_{DRC}=\left({MVYLL}_{1-4}+{MVYLL}_{5-9}+{MVYLL}_{10-14}+{MVYLL}_{15-19}+{MVYLL}_{20-24}+{MVYLL}_{25-29}+\dots +{MVYLL}_{\ge 95}\right) $$

2 $$ {\displaystyle \begin{array}{c}{MVYLL}_i=\sum \limits_{t=1}^n\left\{\left[1/{\left(1+r\right)}^t\right]\times \left[{NHGPC}_{Int\$}\right]\times \left[{EVDD}_i\right]\right\}=\\ {}\left\{\left[1/{\left(1+r\right)}^1\right]\times \left[{NHGPC}_{Int\$}\right]\times \left[{EVDD}_i\right]\right\}+\\ {}\left\{\left[1/{\left(1+r\right)}^2\right]\times \left[{NHGPC}_{Int\$}\right]\times \left[{EVDD}_i\right]\right\}+\dots +\\ {}\left\{\left[1/{\left(1+r\right)}^n\right]\times \left[{NHGPC}_{Int\$}\right]\times \left[{EVDD}_i\right]\right\}\end{array}} $$

Where: 1/(1 + *r*)^*t*^ is the discount factor that converts future GDP losses into today’s dollars; *r* is an interest rate that measures the opportunity cost of lost earnings, i.e. 3% in this study; $$ \sum \limits_{t=1}^n $$ is the summation from year *t* to *n*; *t* is the first year of life lost, and *n* is the final year of the total number of years of life lost per EVD death within an age group, which is obtained by subtracting the age groups average age at death (GAAD) for EVD-related causes from the DRC average life expectancy at birth; *NHGPC*_*Int*$_ is per capita non-health GDP in purchasing power parity (PPP), which is obtained by subtracting per capita current health expenditure (*PCCHE*) from GDP per capita (*GDPPC*_*Int*$_ ); *EVDD*_*i*_ is the number of EVD deaths occurring in age group i, where i = 1 correspond to the age group 1–4 years, i = 2 to the age group 5–9 years, i = 3 to the age group 10–14 years, i = 4 to the age group 15–19 years, i = 5 to the age group 20–24 years, i = 6 to the age group 25–29 years, i = 7 to the age group 30–34 years, i = 8 to the age group 35–39 years, i = 9 to the age group 40–44 years, i = 10 to the age group 45–49 years, i = 11 to the age group 54–54 years, i = 12 to the age group of 55–59 years, i = 13 to the age group 60–64 years, i = 13 to the age group 65–69 years, i = 14 to the age group 70–74 years, i = 15 to the age group 70–74 years, i = 16 to the age group 75–79 years, i = 17 to the age group 80–84 years, i = 18 to the age group 85–89 years, i = 19 to the age group 90–94 years, and i = 20 to the age group 95 years and above in DRC. The base year to which GDP losses occurring in future years were discounted is 2019. The average monetary value per EVD death was obtained by dividing MVYLL_DRC_ by the total number of EVD deaths.

### Sensitivity analysis

Sensitivity analysis is a procedure of assessing the robustness of monetary valuation of years of life lost by considering the effects of uncertainty [15, 27]. In this study, there was uncertainty about the life expectancy at birth and discount rate used. Thus, the algorithm was estimated assuming DRC life expectancy of 60.5 years, the global average life expectancy of 72 years, and Japan female life expectancy of 87 years (the longest in the world) [6]. The algorithm was also re-estimated assuming discount rates of 3, 5 and 10%. The results can be deemed robust if the conclusions remain unchanged [28, 29].

### Data sources

The 1286 EVD-related deaths that occurred between 8 May 2018 and 27 May 2019 were obtained from the DRC Ministry of Health [5]. The distribution of EVD deaths by each of the 20 age groups was estimated using data from the Institute for Health Metrics and Evaluation (IHME) global burden of disease study 2017 database [30]. The DRC life expectancy at birth, Japan female life expectancy, and global average life expectancy were obtained from World Health Statistics 2019 [6]. The current health expenditure per capita of 34 International Dollars (Int$) or Purchasing Power Parity was obtained from the WHO Global Health Expenditure Database [8]. The GDP of Int$791.19 per capita for DRC in 2019 was gotten from IMF World Outlook database [2]. The equations were estimated using Excel Software, Microsoft Corporation, New York.

### Data analysis

The economic model was estimated in 8 steps using Excel Software (Microsoft Corporation, New York):

- *Step 1: Development of analysis algorithm on Excel software*

The equations developed in the HCA analytical framework above were built into a spreadsheet.

- *Step 2: Extraction of EVD mortality data*

The data on the total number of EVD deaths between 8 May 2018 and 27 May 2019 was extracted from the DRC Ministry of Health website [5].

- *Step 3: Distribution of EVD deaths from step 2 across the 20 age groups*

The number of EVD deaths by each of the 20 age groups was obtained by multiplying total number of EVD deaths [5] and relevant proportion [30] (See Additional file 3). For example, the since the total number of EVD deaths was 1286 and the proportion for age group 1–4 years was 0.22857, the number of deaths in age group 1–4 years equals 1286 multiplied by 0.22857, i.e. 294.

- *Step 4: Estimation of undiscounted potential productive years of life lost*

The DRC life expectancy at birth of 60.5 years, global average life expectancy of 72 years, and Japan female life expectancy of 87.1 years (i.e. the highest in the world) were obtained from Annex 1 (part 1) of the World Health Statistics 2019 report [6]. Assuming DRC life expectancy of 60.5 years [6], the i^th^ age group undiscounted potential years of life lost (PYLL) from EVD equals average life expectancy at birth for DRC minus the average age at death for i^th^ age group, where i = 1,2,3,…,20. For example, the PYLL for a death in age group 15–19 equals 43.5 years, i.e. [60.5 – ((15 + 19)/2)]. The undiscounted PYLL for the 20 age groups are contained in Additional file 4.

The undiscounted PYLLs in Additional file 5 were calculated, in a similar manner, assuming the world and the Japan female life expectancies. The PYLL cannot be negative. Thus, for age groups with average age at death greater than DRC life average life expectancy of 60.5 years, their PYLL are assumed to be equal to zero [31].

- Step 5: *Discounting of potentially productive YLL*

The potentially productive YLLs estimated in Step 4 were discounted at a rate (r) of 3% using the following formula: 1/(1 + r)^t^, i.e. the discount factor formula. For example, the first year of life lost would be discounted as follows: [1 × 1/(1 + 0.03)^1^ = 0.97087. The discounted (or present value) for the second YLL would be [1 x [1/(1 + 0.03)^2^] = 0.942596; for third YLL would be [1 x [1/(1 + 0.03)^3^] = 0.915142; and for the n^th^ YLL (e.g. for a premature death in age group 15–19) would be [1 x [1/(1 + 0.03)^43.5^] = 0.276427. Thus, present value of the total years of life lost (PVYLL_i_) from one death in a given i^th^ age group equals the sum of the discount factors multiplied by each undiscounted YLL, i.e.

$$ {PVYLL}_i=\sum \limits_{t=1}^{t=n}{YLL}_i\times \left[\frac{1}{{\left(1+r\right)}^n}\right]. $$

The meanings of *t*, *n* and *r* are as defined earlier under eq. (2). Additional file 6 contains the discounted PYLL from EVD, for all the 20 age groups, assuming DRC life expectancy and 3% discount rate. The discounted potentially productive YLLs in Additional file 7 were calculated, in a similar way, assuming the world and the Japan female life expectancies and a 3% discount rate.

- *Step 6: Estimation of non-health GDP* per capita *(NHGPC*_*Int$*_*)*

The data on GDP per capita of Int$791.19 was obtained from the IMF World Outlook Database [2]; and current health expenditure per capita of Int$34 from WHO Global Health Expenditure Database [8]. The NHGPC_Int$_ was the difference between GDP per capita and current health expenditure per capita for DRC, i.e. Int$791.19 – Int$34 = Int$757.19 per person.

- *Step 7: Calculation of MVYLL per age group*

The algorithm developed in Step 1 was estimated assuming the DRC life expectancy at birth and 3% discount rate. The information on number of deaths by age group in Additional file 3; discounted potential years of life lost from EVD assuming DRC life expectancy in Additional file 6; and non-health GDP per capita for DRC were used in calculation of MVYLL for each age group.

For example, MVYLL for 1–4 years’ age group was the product of non-health GDP per capita (Int$757.19), discounted years of life lost per death in the age group (25.02470783) and number of EVD deaths in the age group (294). Therefore, MVYLL_1–4_ = 757.19 × 25.02470783 × 294 = Int$ 5,570,847. The MVYLL for the other age groups was calculated in a similar manner.

- *Step 8: Conduct of sensitivity analysis*
  - *Step 8.1: Sensitivity to discount rate*

Equations (1) to (20) were re-estimated with 5% (see Additional file 8) and 10% (see Additional file 9) discount rates to measure the sensitivity of the monetary value of years of life lost. For example, at the DRC life expectancy and 5% discount rate, the MVYLL_1–4_ = 757.19 × 17.98101571 × 294 = Int$4,002,823. At the world life expectancy and 5% discount rate, the MVYLL_1–4_ = 757.19 × 18.8195417 × 294 = Int$4,189,491. At the Japanese Female life expectancy and 5% discount rate, the MVYLL_1–4_ = 757.19 × 19.43217937 × 294 = Int$ 4,325,872.

At the DRC life expectancy and 10% discount rate, the MVYLL_1–4_ = 757.19 × 9.886618082 × 294 = Int$ 2,200,898. At the world life expectancy and 10% discount rate, the MVYLL_1–4_ = 757.19 × 9.96026033 × 294 = Int$2,217,292. At the Japanese Female life expectancy and 10% discount rate, the MVYLL_1–4_ = 757.19 × 9.990486639 × 294 = Int$2,224,021. The sensitivity analysis of MVYLL to changes in discount rates for the other age groups was calculated in the same way.

- *Step 8.2: Sensitivity to life expectancy*

In order to determine the impact of life expectancy on MVYLL estimates, the equations were re-estimated alternately assuming the world life expectancy of 72 years (Additional file 7) and the Japanese female life expectancy of 87 years (which is the highest in the world) (Additional file 7). At the world life expectancy of 72 years and 3% discount rate, the MVYLL_1–4_ = 757.19 × 27.33100549 × 294 = Int$ 6,084,261. At the Japanese Female life expectancy of 87 years and 3% discount rate, the MVYLL_1–4_ = 757.19 × 29.4806675 × 294 = Int$ 6,562,805. The sensitivity analysis of MVYLL to changes in life expectancy for the other age groups was estimated in the same manner.

## Results

Table 1 provides a breakdown of the monetary value of potential years of life lost from Ebola Virus Disease in DRC discounted at a 3% rate. The 1286 deaths resulted in total MVYLL of Int$17,761,539; Int$21,428,624; and Int$25,270,037 assuming DRC life expectancy of 60.5 years, the average global life expectancy of 72 years and Japan female life expectancy of 87 years, respectively. The average MVYLL assuming DRC, global and Japan female life expectancies was Int$13,801, Int$16,650 and Int$19,635 per death, respectively.

**Table 1:** Monetary value of years of life lost from Ebola Virus Disease in DRC discounted at 3% (2019 Int$ or PPP)

Age Group	MVYLL assuming DRC average life expectancy & 3% discount rate^1^	MVYLL assuming global average life expectancy of 72 years & 3% discount rate^2^	MVYLL assuming Japan female life expectancy of 87 years & 3% discount rate^3^
1–4	5,570,847	6,084,261	6,562,805
5–9	2,368,557	2,586,846	2,790,308
10–14	1,193,753	1,303,770	1,406,315
15–19	826,429	912,300	992,338
20–24	656,266	740,328	818,681
25–29	560,029	649,786	733,448
30–34	508,526	612,580	709,567
35–39	4,962,665	6,296,454	7,539,658
40–44	412,142	563,968	705,482
45–49	342,131	527,402	700,091
50–54	247,614	473,133	683,336
55–59	112,582	361,571	593,650
60–64	0	226,064	461,477
65–69	0	90,160	292,892
70–74	0	0	171,746
75–79	0	0	83,967
80–84	0	0	24,274
85–89	0	0	0
90–94	0	0	0
95+	0	0	0
TOTAL	17,761,539	21,428,624	25,270,037

^1^DRC life expectancy and 3% discount rate; ^2^Global average life expectancy of 87 years and 3% discount rate; ^3^Japan female life expectancy of 87 years and 3% discount rate

Out of the Int$17,761,539, about 31.4% occurred among 1–4-year-old; 13.3% among 5–9-year-old; 6.7% among 10–14-year-old; 4.7% among 15–19-year-old; 3.7% among 20–24-year-old; 3.2% among 25–29-year-old; 2.9% among 30–34-year-old; 27.9% among 35–39-year-old; 2.3% among 40–44-year-old; 1.9% among 45–49-year-old; 1.4% among 50–54-year-old; and 0.6% among 55–59-year-old. Thus, 48.6% of the MVYLL occurred among adults aged between 15 years and 59 years; which is the most productive age bracket.

Use of the Japanese female life expectancy at birth of 87 years, holding discount rate constant at 3%, increased the MVYLL by Int$ 7,508,498 (42.3%). Utilization of the average global life expectancy at birth of 72 years, holding discount rate constant at 3%, increased the MVYLL by Int$ 3,667,085 (20.6%).

Table 2 presents an age-group break-down of MVYLL from EVD discounted at a 5% rate. The application of 5% discount rate holding life expectancy constant at 60.5 years, 72 years and 87 years reduced the MVYLL by Int$ 4,252,785 (− 23.94%), Int$ 5,806,233 (− 27.1%), and 7,737,915 (− 30.6%) respectively.

**Table 2:** Monetary value of years of life lost from Ebola Virus Disease in DRC discounted at 5% (2019 Int$ or PPP)

Age Group	MVYLL assuming DRC life expectancy 60.5 years & 5% discount rate^1^	MVYLL assuming global average life expectancy of 72 years & 5% discount rate^2^	MVYLL assuming japan female life expectancy of 87 years & 5% discount rate^3^
1–4	4,002,823	4,189,491	4,325,872
5–9	1,701,881	1,781,246	1,839,231
10–14	857,748	897,748	926,973
15–19	601,833	634,909	659,074
20–24	489,635	525,282	551,326
25–29	429,139	471,042	501,657
30–34	401,263	454,744	493,818
35–39	4,043,451	4,798,172	5,349,580
40–44	347,734	442,315	511,417
45–49	299,806	426,872	519,708
50–54	226,043	396,323	520,732
55–59	107,398	314,375	465,595
60–64	0	204,639	373,513
65–69	0	85,234	245,343
70–74	0	0	149,328
75–79	0	0	76,009
80–84	0	0	22,948
85–89	0	0	0
90–94	0	0	0
95+	0	0	0
TOTAL	13,508,754	15,622,391	17,532,122

^1^DRC life expectancy 60.5 years and 5% discount rate; ^2^Global average life expectancy of 72 years and 5% discount rate; ^3^Japan female life expectancy of 87 years and 5% discount rate; minimum working age in DRC is 15 years, and thus, PYLL below 14 years were value at zero

Table 3 presents an age-group break-down of MVYLL from EVD discounted at a 10% rate. The application of 10% discount rate holding life expectancies constant at 60.5 years, 72 years and 87 years reduced the MVYLL by Int$ 9,658,195 (−54.4%), Int$ 12,537,911 (−58.5%), and Int$ 15,792,925 (−62.5%) respectively.

**Table 3:** Monetary value of years of life lost from Ebola Virus Disease in DRC discounted at 10% (2019 Int$ or PPP)

Age Group	MVYLL assuming DRC life expectancy & 10% discount rate^1^	MVYLL assuming global average life expectancy of 72 years & 10% discount rate^3^	MVYLL assuming Japan female life expectancy of 87 years & 10% discount rate^2^
1–4	2,200,898	2,217,292	2,224,021
5–9	935,756	942,726	945,587
10–14	471,621	475,134	476,576
15–19	335,593	338,933	340,304
20–24	280,739	285,281	287,145
25–29	254,643	261,381	264,146
30–34	248,310	259,161	263,615
35–39	2,632,822	2,826,052	2,905,363
40–44	240,686	271,243	283,785
45–49	223,119	274,922	296,184
50–54	183,148	270,748	306,703
55–59	96,008	230,370	285,519
60–64	0	162,841	240,557
65–69	0	74,629	167,606
70–74	0	–	109,426
75–79	0	–	60,484
80–84	0	–	20,092
85–89	0	–	0
90–94	0	–	0
95+	0	–	0
TOTAL	8,103,344	8,890,713	9,477,112

^1^DRC life expectancy 60.5 years and 10% discount rate; ^2^Japan female life expectancy of 87 years & 10% discount rate; ^3^Global average life expectancy of 72 years & 10% discount rate; minimum working age in DRC is 15 years, and thus, PYLL below 14 years were value at zero

## Discussion

This study has estimated the monetary value of human lives lost due to EVD outbreak between 8 May 2018 and 27 May 2019 in DRC. The 1286 EVD-related deaths resulted in total MVYLL of Int$17,761,539; which translates to Int$13,801 per death. The main drivers of MVYLL estimates were the life expectancy and discount rate used.

How does this MVYLL per EVD-related death in DRC compare to similar studies conducted in the past in other African countries? Kirigia et al [11] estimated productivity loss per EVD death to be Int$ 17,473 for Guinea, Int$ 11,283 for Liberia, Int$ 25,126 for Mali, Int$ 47,364 for Nigeria and Int$ 14,633 for Sierra Leone. Thus, MVYLL per EVD death in DRC is lower than that of Guinea, Mali, Nigeria and Sierra Leone due to lower per capita GDP.

How does this MVYLL per EVD-related death compare to that of other diseases? Kirigia et al [19] estimated the African Region average grand total non-health GDP loss to be Int$ 30,203 per maternal death; and Int$ 16,397 per maternal death in 2010 for low-income countries. Another study estimated the African Region low-income countries discounted value of future non-health GDP loss due to maternal deaths in 2013 at Int$19,822 per maternal death [20]. Kirigia and Muthuri [23] appraised the African Region average grand total non-health GDP loss to be Int$66,872 per tuberculosis death; and Int$21,513 per tuberculosis death in 2014 for low-income countries. The expected future productivity loss per child death in the WHO African Region in 2013 was estimated to be Int$50,494; and Int$ 25,508 per child death for low-income countries [21]. In 2015 the mean value of human life lost per NTD death in the African Continent was Int$ 37,489; and Int$ 37,489 per human life in low-income countries [22]. In 2012 the WHO African Region average grand total non-health GDP loss was Int$ 21,985 per NCD death; and Int$ 9096 per NCD death for low-income countries [24]. Therefore, the estimate of MVYLL per EVD death in DRC is lower than the indirect cost per maternal death, tuberculosis death, child death and NTD death in Africa. However, MVYLL per EVD death in DRC was larger than the indirect cost per NCD death in low-income countries in Africa. Given the virulent nature of EVD and its multi-sectoral negative consequences, concerted national, continental and global effort should be made to eradicate EVD in the DRC.

### Limitations of the study

The study reported in this paper has four main limitations. First, it omits the cost of productivity time lost among those diagnosed with EVD but do not die; health system costs, including vaccination, tracing cases, diagnostic tests, transport of health workers and patients (suspected and actual), plus building and operating treatment centres (including health workforce, medicines, medical devices); and intangible costs of pain, grief, mental anguish and social stigma to patients and family members [32]. In addition, this study does not include the negative impact of fear and stigmatisation of EVD on various socioeconomic activities, e.g. aviation, education (schooling), mining, tourism (hospitality), etc. [33–35].

Second, strictly applied the HCA would attach a zero monetary value on the years of life lost among the children below minimum working age of 15 years; unemployed adults; full-time homemakers; and retired people [16, 36]. Even though their contribution does not feature in GDP calculations, the last three categories of people make an important non-monetized contribution to the society, and thus, we valued their YLL at the net non-health GDP per capita for DRC. With regard to deaths among children, only the YLL above the minimum working age of 15 years were valued.

Third, HCA is “..implicitly based upon the maximization of society’s present and future production” (p. 556) [36]. Maximization of economic production is not the only reason for combatting EVD. There are other reasons such as restoring patients to health so that they can flourish, enjoy leisure, and realize their inalienable right to health [37]. In addition, society desires to combat EVD to reduce (or alleviate) fear and anxiety in the population, and pave way for unimpeded international flow of goods and people.

Fourth, the HCA does not weigh the costs and benefits of alternative interventions into EVD [38, 39]. Thus, its results should not be used as a basis for priority setting, but primarily for advocacy for increased investments into the national health system (including disease surveillance system) and other systems that address the social determinants of health.

## Conclusion

The ongoing EVD outbreak in the DRC has led to the substantive monetary value of years of life lost. Therefore, there is urgent need for DRC government and development partners to disburse adequate resources to strengthen national health system (including disease surveillance system) and other systems that address social determinants of health (education, water, sanitation, hygiene, cultural practices, physical security) to end recurrence of EVD outbreaks [40].

In addition, there is a necessity for conducting economic evaluations that identify, assess and compare the costs and consequences associated with alternative preventive and treatment interventions for EVD. Unlike, the evidence presented in this paper which is primarily for use in advocacy, economic evaluation evidence would guide priority-setting, decision-making and planning of prevention and control actions for combatting EVD.

## Additional files

Additional file 1: Epidemiological situation of Ebola Virus Disease in DRC as at 27 May 2019. (DOCX 12 kb)

Additional file 2: Equations used to estimate the DRC’s discounted total non-health GDP loss attributable to EVD deaths (MVYLL_DRC_). (DOCX 91 kb)

Additional file 3: Age distribution of EVD deaths in DRC. (DOCX 12 kb)

Additional file 4 Undiscounted potential years of life lost from EVD assuming DRC life expectancy. (DOCX 12 kb)

Additional file 5: Undiscounted potential years of life lost from EVD assuming world’s and Japanese Female life expectancies. (DOCX 13 kb)

Additional file 6: Discounted potential years of life lost from EVD assuming DRC life expectancy and 3% discount rate. (DOCX 12 kb)

Additional file 7: Discounted potential years of life lost from EVD assuming world’s and Japan Female life expectancies and a 3% discount rate. (DOCX 13 kb)

Additional file 8: Discounted potential years of life lost from EVD assuming the DRC, the world and Japanese female life expectancies and a 5% discount rate. (DOCX 13 kb)

Additional file 9: Discounted potential years of life lost from EVD assuming the DRC, the World and the Japan female life expectancies and a 10% discount rate. (DOCX 13 kb)

## Data Availability

All data generated or analysed during this study are included in this published article and its supplementary information files.

## Abbreviations

CHE: Current health expenditure
DRC: Democratic Republic of the Congo
EVD: Ebola Virus Disease
EVDD_i_: Number of Ebola Virus Disease deaths per i^th^ age group
EXT: External health expenditure
GDP: Gross domestic product
GGHE-D: Domestic general government health expenditure
HCA: Human capital approach
IHME: Institute of Health Metrics and Evaluation
IMF: International Monetary Fund
Int$: International Dollars or Purchasing Power Parity (PPP)
MVYLL: Monetary value of years of life lost
MVYLL_= > 95_: Monetary value of potential years of life lost among those aged 95 years and above
MVYLL_10–14_: Monetary value of potential years of life lost among those aged 10–14
MVYLL_1–4_: Monetary value of potential years of life lost among those aged 1–4
MVYLL_15–19_: Monetary value of potential years of life lost among those aged 15–19
MVYLL_20–24_: Monetary value of potential years of life lost among those aged 20–24
MVYLL_25–29_: Monetary value of potential years of life lost among those aged 25–29
MVYLL_30–34_: Monetary value of potential years of life lost among those aged 30–34
MVYLL_35–39_: Monetary value of potential years of life lost among those aged 35–39
MVYLL_40–44_: Monetary value of potential years of life lost among those aged 40–44
MVYLL_45–49_: Monetary value of potential years of life lost among those aged 45–49
MVYLL_50–54_: Monetary value of potential years of life lost among those aged 50–54
MVYLL_55–59_: Monetary value of potential years of life lost among those aged 55–59
MVYLL_5–9_: Monetary value of potential years of life lost among those aged 5–9
MVYLL_60–64_: Monetary value of potential years of life lost among those aged 60–64
MVYLL_65–69_: Monetary value of potential years of life lost among those aged 65–69
MVYLL_70–74_: Monetary value of potential years of life lost among those aged 70–74
MVYLL_75–79_: Monetary value of potential years of life lost among those aged 75–79
MVYLL_80–84_: Monetary value of potential years of life lost among those aged 80–84
MVYLL_85–89_: Monetary value of potential years of life lost among those aged 85–89
MVYLL_90–94_: Monetary value of potential years of life lost among those aged 90–94
NCD: Non-communicable disease
NHGPC_Int$_: Per capita non-health GDP in purchasing power parity
NTD: Neglected tropical disease
OOPS: Out-of-pocket expenditure
PVT-D: Domestic private health expenditure
PYLL: Potential Years of Life Lost
r: Interest rate that measures the opportunity cost of lost earnings
SDG3: United Nations Sustainable Development Goal
UHC: Universal health coverage
US$: United States Dollar
WHO: World Health Organization
