# Supplementary material for: The monetary value of human lives lost through Ebola virus disease in the Democratic Republic of Congo in 2019
Source: BMC Public Health. 2019 Sep 3;19:1218. doi: 10.1186/s12889-019-7542-2 (PMC6724278; doi:10.1186/s12889-019-7542-2)
Supplement: Supplementary file 1 — Epidemiological situation of Ebola Virus Disease in DRC as at 27 May 2019. (DOCX 12 kb) [file 12889_2019_7542_MOESM1_ESM.docx]

| **Additional File 1: Epidemiological situation of Ebola Virus Disease in DRC as at 27 May 2019** | | | |
| --- | --- | --- | --- |
| **Provinces** | **Health Zones** | **Total EVD cases** | **EVD related Deaths** |
| North-Kivu | Alimbongo | 1 | 0 |
|  | Beni | 314 | 184 |
|  | Biena | 9 | 10 |
|  | Butembo | 217 | 233 |
|  | Goma | 0 | 0 |
|  | Kalunguta | 116 | 61 |
|  | Katwa | 588 | 401 |
|  | Kayna | 8 | 5 |
|  | Kyondo | 21 | 15 |
|  | Lubero | 13 | 4 |
|  | Mabalako | 210 | 153 |
|  | Mangurujipa | 11 | 5 |
|  | Masereka | 43 | 20 |
|  | Musienene | 56 | 25 |
|  | Mutwuanga | 5 | 3 |
|  | Oicha | 41 | 20 |
|  | Vuhovi | 96 | 42 |
| Ituri | Bunia | 1 | 1 |
|  | Komanda | 37 | 19 |
|  | Mambasa | 0 | 0 |
|  | Mandima | 135 | 82 |
|  | Nyankunde | 1 | 1 |
|  | Rwampara | 1 | 1 |
|  | Tchomia | 2 | 1 |
| **TOTAL** |  | **1926** | **1286** |

Source: DRC Ministry of Health [5].
