# Supplementary material for: The monetary value of human lives lost through Ebola virus disease in the Democratic Republic of Congo in 2019
Source: BMC Public Health. 2019 Sep 3;19:1218. doi: 10.1186/s12889-019-7542-2 (PMC6724278; doi:10.1186/s12889-019-7542-2)
Supplement: Supplementary file 2 — Equations used to estimate the DRC’s discounted total non-health GDP loss attributable to EVD deaths (MVYLLDRC). (DOCX 91 kb) [file 12889_2019_7542_MOESM2_ESM.docx]

**Additional File 2: Equations used to estimate the DRC’s discounted total non-health GDP loss attributable to EVD deaths (MVYLL_DRC_)**

$$MVYLL_{DRC}= \left(\begin{matrix} MVYLL_{1-4} +MVYLL_{5-9} + MVYLL_{10-14} + MVYLL_{15-19} + \\ MVYLL_{20-24} + MVYLL_{25-29} +...+ MVYLL_{\geq95} \end{matrix}\right) ....\left(1\right)$$

$$\begin{matrix} MVYLL_{1-4}= \sum_{t=1}^{n} \left\{\left[1/\left(1+r\right)^{t}\right]\right.\times\left[NHGPC_{Int\$}\right]\times\left.\left[EVDD_{1-4}\right]\right\} = \\ \left\{\left[1/\left(1+r\right)^{1}\right]\right.\times\left[NHGPC_{Int\$}\right]\times\left.\left[EVDD_{1-4}\right]\right\}+ \\ \left\{\left[1/\left(1+r\right)^{2}\right]\right.\times\left[NHGPC_{Int\$}\right]\times\left.\left[EVDD_{1-4}\right]\right\}+...+ \\ \left\{\left[1/\left(1+r\right)^{n}\right]\right.\times\left[NHGPC_{Int\$}\right]\times\left.\left[EVDD_{1-4}\right]\right\} ....................................\left(2\right) \end{matrix}$$

$$\begin{matrix} MVYLL_{5-9}= \sum_{t=1}^{n} \left\{\left[1/\left(1+r\right)^{t}\right]\right.\times\left[NHGPC_{Int\$}\right]\times\left.\left[EVDD_{5-9}\right]\right\} = \\ \left\{\left[1/\left(1+r\right)^{1}\right]\right.\times\left[NHGPC_{Int\$}\right]\times\left.\left[EVDD_{5-9}\right]\right\}+ \\ \left\{\left[1/\left(1+r\right)^{2}\right]\right.\times\left[NHGPC_{Int\$}\right]\times\left.\left[EVDD_{5-9}\right]\right\}+...+ \\ \left\{\left[1/\left(1+r\right)^{n}\right]\right.\times\left[NHGPC_{Int\$}\right]\times\left.\left[EVDD_{5-9}\right]\right\} ....................................\left(3\right) \end{matrix}$$

$$\begin{matrix} MVYLL_{10-14}= \sum_{t=1}^{n} \left\{\left[1/\left(1+r\right)^{t}\right]\right.\times\left[NHGPC_{Int\$}\right]\times\left.\left[EVDD_{10-14}\right]\right\} = \\ \left\{\left[1/\left(1+r\right)^{1}\right]\right.\times\left[NHGPC_{Int\$}\right]\times\left.\left[EVDD_{10-14}\right]\right\}+ \\ \left\{\left[1/\left(1+r\right)^{2}\right]\right.\times\left[NHGPC_{Int\$}\right]\times\left.\left[EVDD_{10-14}\right]\right\}+...+ \\ \left\{\left[1/\left(1+r\right)^{n}\right]\right.\times\left[NHGPC_{Int\$}\right]\times\left.\left[EVDD_{10-14}\right]\right\} ....................................\left(4\right) \end{matrix}$$

$$\begin{matrix} MVYLLs_{15-19}= \sum_{t=1}^{n} \left\{\left[1/\left(1+r\right)^{t}\right]\right.\times\left[NHGPC_{Int\$}\right]\times\left.\left[EVDD_{15-19}\right]\right\} = \\ \left\{\left[1/\left(1+r\right)^{1}\right]\right.\times\left[NHGPC_{Int\$}\right]\times\left.\left[EVDD_{15-19}\right]\right\}+ \\ \left\{\left[1/\left(1+r\right)^{2}\right]\right.\times\left[NHGPC_{Int\$}\right]\times\left.\left[EVDD_{15-19}\right]\right\}+...+ \\ \left\{\left[1/\left(1+r\right)^{n}\right]\right.\times\left[NHGPC_{Int\$}\right]\times\left.\left[EVDD_{15-19}\right]\right\} ....................................\left(5\right) \end{matrix}$$

$$\begin{matrix} MVYLL_{20-24}= \sum_{t=1}^{n} \left\{\left[1/\left(1+r\right)^{t}\right]\right.\times\left[NHGPC_{Int\$}\right]\times\left.\left[EVDD_{20-24}\right]\right\} = \\ \left\{\left[1/\left(1+r\right)^{1}\right]\right.\times\left[NHGPC_{Int\$}\right]\times\left.\left[EVDD_{20-24}\right]\right\}+ \\ \left\{\left[1/\left(1+r\right)^{2}\right]\right.\times\left[NHGPC_{Int\$}\right]\times\left.\left[EVDD_{20-24}\right]\right\}+...+ \\ \left\{\left[1/\left(1+r\right)^{n}\right]\right.\times\left[NHGPC_{Int\$}\right]\times\left.\left[EVDD_{20-24}\right]\right\} ....................................\left(6\right) \end{matrix}$$

$$\begin{matrix} MVYLL_{25-29}= \sum_{t=1}^{n} \left\{\left[1/\left(1+r\right)^{t}\right]\right.\times\left[NHGPC_{Int\$}\right]\times\left.\left[EVDD_{25-29}\right]\right\} = \\ \left\{\left[1/\left(1+r\right)^{1}\right]\right.\times\left[NHGPC_{Int\$}\right]\times\left.\left[EVDD_{25-29}\right]\right\}+ \\ \left\{\left[1/\left(1+r\right)^{2}\right]\right.\times\left[NHGPC_{Int\$}\right]\times\left.\left[EVDD_{25-29}\right]\right\}+...+ \\ \left\{\left[1/\left(1+r\right)^{n}\right]\right.\times\left[NHGPC_{Int\$}\right]\times\left.\left[EVDD_{25-29}\right]\right\} ....................................\left(7\right) \end{matrix}$$

$$\begin{matrix} MVYLL_{30-34}= \sum_{t=1}^{n} \left\{\left[1/\left(1+r\right)^{t}\right]\right.\times\left[NHGPC_{Int\$}\right]\times\left.\left[EVDD_{30-34}\right]\right\} = \\ \left\{\left[1/\left(1+r\right)^{1}\right]\right.\times\left[NHGPC_{Int\$}\right]\times\left.\left[EVDD_{30-34}\right]\right\}+ \\ \left\{\left[1/\left(1+r\right)^{2}\right]\right.\times\left[NHGPC_{Int\$}\right]\times\left.\left[EVDD_{30-34}\right]\right\}+...+ \\ \left\{\left[1/\left(1+r\right)^{n}\right]\right.\times\left[NHGPC_{Int\$}\right]\times\left.\left[EVDD_{30-34}\right]\right\} ....................................\left(8\right) \end{matrix}$$

$$\begin{matrix} MVYLL_{35-39}= \sum_{t=1}^{n} \left\{\left[1/\left(1+r\right)^{t}\right]\right.\times\left[NHGPC_{Int\$}\right]\times\left.\left[EVDD_{35-39}\right]\right\} = \\ \left\{\left[1/\left(1+r\right)^{1}\right]\right.\times\left[NHGPC_{Int\$}\right]\times\left.\left[EVDD_{35-39}\right]\right\}+ \\ \left\{\left[1/\left(1+r\right)^{2}\right]\right.\times\left[NHGPC_{Int\$}\right]\times\left.\left[EVDD_{35-39}\right]\right\}+...+ \\ \left\{\left[1/\left(1+r\right)^{n}\right]\right.\times\left[NHGPC_{Int\$}\right]\times\left.\left[EVDD_{35-39}\right]\right\} ....................................\left(9\right) \end{matrix}$$

$$\begin{matrix} MVYLL_{40-44}= \sum_{t=1}^{n} \left\{\left[1/\left(1+r\right)^{t}\right]\right.\times\left[NHGPC_{Int\$}\right]\times\left.\left[EVDD_{40-44}\right]\right\} = \\ \left\{\left[1/\left(1+r\right)^{1}\right]\right.\times\left[NHGPC_{Int\$}\right]\times\left.\left[EVDD_{40-44}\right]\right\}+ \\ \left\{\left[1/\left(1+r\right)^{2}\right]\right.\times\left[NHGPC_{Int\$}\right]\times\left.\left[EVDD_{40-44}\right]\right\}+...+ \\ \left\{\left[1/\left(1+r\right)^{n}\right]\right.\times\left[NHGPC_{Int\$}\right]\times\left.\left[EVDD_{40-44}\right]\right\} ....................................\left(10\right) \end{matrix}$$

$$\begin{matrix} MVYLL_{45-49}= \sum_{t=1}^{n} \left\{\left[1/\left(1+r\right)^{t}\right]\right.\times\left[NHGPC_{Int\$}\right]\times\left.\left[EVDD_{45-49}\right]\right\} = \\ \left\{\left[1/\left(1+r\right)^{1}\right]\right.\times\left[NHGPC_{Int\$}\right]\times\left.\left[EVDD_{45-49}\right]\right\}+ \\ \left\{\left[1/\left(1+r\right)^{2}\right]\right.\times\left[NHGPC_{Int\$}\right]\times\left.\left[EVDD_{45-49}\right]\right\}+...+ \\ \left\{\left[1/\left(1+r\right)^{n}\right]\right.\times\left[NHGPC_{Int\$}\right]\times\left.\left[EVDD_{45-49}\right]\right\} ....................................\left(11\right) \end{matrix}$$

$$\begin{matrix} MVYLL_{50-54}= \sum_{t=1}^{n} \left\{\left[1/\left(1+r\right)^{t}\right]\right.\times\left[NHGPC_{Int\$}\right]\times\left.\left[EVDD_{50-54}\right]\right\} = \\ \left\{\left[1/\left(1+r\right)^{1}\right]\right.\times\left[NHGPC_{Int\$}\right]\times\left.\left[EVDD_{50-54}\right]\right\}+ \\ \left\{\left[1/\left(1+r\right)^{2}\right]\right.\times\left[NHGPC_{Int\$}\right]\times\left.\left[EVDD_{50-54}\right]\right\}+...+ \\ \left\{\left[1/\left(1+r\right)^{n}\right]\right.\times\left[NHGPC_{Int\$}\right]\times\left.\left[EVDD_{50-54}\right]\right\} ....................................\left(12\right) \end{matrix}$$

$$\begin{matrix} MVYLL_{55-59}= \sum_{t=1}^{n} \left\{\left[1/\left(1+r\right)^{t}\right]\right.\times\left[NHGPC_{Int\$}\right]\times\left.\left[EVDD_{55-59}\right]\right\} = \\ \left\{\left[1/\left(1+r\right)^{1}\right]\right.\times\left[NHGPC_{Int\$}\right]\times\left.\left[EVDD_{55-59}\right]\right\}+ \\ \left\{\left[1/\left(1+r\right)^{2}\right]\right.\times\left[NHGPC_{Int\$}\right]\times\left.\left[EVDD_{55-59}\right]\right\}+...+ \\ \left\{\left[1/\left(1+r\right)^{n}\right]\right.\times\left[NHGPC_{Int\$}\right]\times\left.\left[EVDD_{55-59}\right]\right\} ....................................\left(13\right) \end{matrix}$$

$$\begin{matrix} MVYLL_{60-64}= \sum_{t=1}^{n} \left\{\left[1/\left(1+r\right)^{t}\right]\right.\times\left[NHGPC_{Int\$}\right]\times\left.\left[EVDD_{60-64}\right]\right\} = \\ \left\{\left[1/\left(1+r\right)^{1}\right]\right.\times\left[NHGPC_{Int\$}\right]\times\left.\left[EVDD_{60-64}\right]\right\}+ \\ \left\{\left[1/\left(1+r\right)^{2}\right]\right.\times\left[NHGPC_{Int\$}\right]\times\left.\left[EVDD_{60-64}\right]\right\}+...+ \\ \left\{\left[1/\left(1+r\right)^{n}\right]\right.\times\left[NHGPC_{Int\$}\right]\times\left.\left[EVDD_{60-64}\right]\right\} ....................................\left(14\right) \end{matrix}$$

$$\begin{matrix} MVYLL_{65-69}= \sum_{t=1}^{n} \left\{\left[1/\left(1+r\right)^{t}\right]\right.\times\left[NHGPC_{Int\$}\right]\times\left.\left[EVDD_{65-69}\right]\right\} = \\ \left\{\left[1/\left(1+r\right)^{1}\right]\right.\times\left[NHGPC_{Int\$}\right]\times\left.\left[EVDD_{65-69}\right]\right\}+ \\ \left\{\left[1/\left(1+r\right)^{2}\right]\right.\times\left[NHGPC_{Int\$}\right]\times\left.\left[EVDD_{65-69}\right]\right\}+...+ \\ \left\{\left[1/\left(1+r\right)^{n}\right]\right.\times\left[NHGPC_{Int\$}\right]\times\left.\left[EVDD_{65-69}\right]\right\} ....................................\left(15\right) \end{matrix}$$

$$\begin{matrix} MVYLL_{70-74}= \sum_{t=1}^{n} \left\{\left[1/\left(1+r\right)^{t}\right]\right.\times\left[NHGPC_{Int\$}\right]\times\left.\left[EVDD_{70-74}\right]\right\} = \\ \left\{\left[1/\left(1+r\right)^{1}\right]\right.\times\left[NHGPC_{Int\$}\right]\times\left.\left[EVDD_{70-74}\right]\right\}+ \\ \left\{\left[1/\left(1+r\right)^{2}\right]\right.\times\left[NHGPC_{Int\$}\right]\times\left.\left[EVDD_{70-74}\right]\right\}+...+ \\ \left\{\left[1/\left(1+r\right)^{n}\right]\right.\times\left[NHGPC_{Int\$}\right]\times\left.\left[EVDD_{70-74}\right]\right\} ....................................\left(16\right) \end{matrix}$$

$$\begin{matrix} MVYLL_{75-79}= \sum_{t=1}^{n} \left\{\left[1/\left(1+r\right)^{t}\right]\right.\times\left[NHGPC_{Int\$}\right]\times\left.\left[EVDD_{75-79}\right]\right\} = \\ \left\{\left[1/\left(1+r\right)^{1}\right]\right.\times\left[NHGPC_{Int\$}\right]\times\left.\left[EVDD_{75-79}\right]\right\}+ \\ \left\{\left[1/\left(1+r\right)^{2}\right]\right.\times\left[NHGPC_{Int\$}\right]\times\left.\left[EVDD_{75-79}\right]\right\}+...+ \\ \left\{\left[1/\left(1+r\right)^{n}\right]\right.\times\left[NHGPC_{Int\$}\right]\times\left.\left[EVDD_{75-79}\right]\right\} ....................................\left(17\right) \end{matrix}$$

$$\begin{matrix} MVYLL_{80-84}= \sum_{t=1}^{n} \left\{\left[1/\left(1+r\right)^{t}\right]\right.\times\left[NHGPC_{Int\$}\right]\times\left.\left[EVDD_{80-84}\right]\right\} = \\ \left\{\left[1/\left(1+r\right)^{1}\right]\right.\times\left[NHGPC_{Int\$}\right]\times\left.\left[EVDD_{80-84}\right]\right\}+ \\ \left\{\left[1/\left(1+r\right)^{2}\right]\right.\times\left[NHGPC_{Int\$}\right]\times\left.\left[EVDD_{80-84}\right]\right\}+...+ \\ \left\{\left[1/\left(1+r\right)^{n}\right]\right.\times\left[NHGPC_{Int\$}\right]\times\left.\left[EVDD_{80-84}\right]\right\} ....................................\left(18\right) \end{matrix}$$

$$\begin{matrix} MVYLL_{85-89}= \sum_{t=1}^{n} \left\{\left[1/\left(1+r\right)^{t}\right]\right.\times\left[NHGPC_{Int\$}\right]\times\left.\left[EVDD_{85-89}\right]\right\} = \\ \left\{\left[1/\left(1+r\right)^{1}\right]\right.\times\left[NHGPC_{Int\$}\right]\times\left.\left[EVDD_{85-89}\right]\right\}+ \\ \left\{\left[1/\left(1+r\right)^{2}\right]\right.\times\left[NHGPC_{Int\$}\right]\times\left.\left[EVDD_{85-89}\right]\right\}+...+ \\ \left\{\left[1/\left(1+r\right)^{n}\right]\right.\times\left[NHGPC_{Int\$}\right]\times\left.\left[EVDD_{85-89}\right]\right\} ....................................\left(19\right) \end{matrix}$$

$$\begin{matrix} MVYLL_{90-94}= \sum_{t=1}^{n} \left\{\left[1/\left(1+r\right)^{t}\right]\right.\times\left[NHGPC_{Int\$}\right]\times\left.\left[EVDD_{90-94}\right]\right\} = \\ \left\{\left[1/\left(1+r\right)^{1}\right]\right.\times\left[NHGPC_{Int\$}\right]\times\left.\left[EVDD_{90-94}\right]\right\}+ \\ \left\{\left[1/\left(1+r\right)^{2}\right]\right.\times\left[NHGPC_{Int\$}\right]\times\left.\left[EVDD_{90-94}\right]\right\}+...+ \\ \left\{\left[1/\left(1+r\right)^{n}\right]\right.\times\left[NHGPC_{Int\$}\right]\times\left.\left[EVDD_{90-94}\right]\right\} ....................................\left(20\right) \end{matrix}$$

$$\begin{matrix} MVYLL_{\geq95}= \sum_{t=1}^{n} \left\{\left[1/\left(1+r\right)^{t}\right]\right.\times\left[NHGPC_{Int\$}\right]\times\left.\left[EVDD_{\geq95}\right]\right\} = \\ \left\{\left[1/\left(1+r\right)^{1}\right]\right.\times\left[NHGPC_{Int\$}\right]\times\left.\left[EVDD_{\geq95}\right]\right\}+ \\ \left\{\left[1/\left(1+r\right)^{2}\right]\right.\times\left[NHGPC_{Int\$}\right]\times\left.\left[EVDD_{\geq95}\right]\right\}+...+ \\ \left\{\left[1/\left(1+r\right)^{n}\right]\right.\times\left[NHGPC_{Int\$}\right]\times\left.\left[EVDD_{\geq95}\right]\right\} ....................................\left(21\right). \end{matrix}$$

Where: $1/\left(1+r\right)^{t}$ is the discount factor that converts future GDP losses into today’s dollars; $r$ is an interest rate that measures the opportunity cost of lost earnings, i.e. 3% in this study; is the summation from year $t$ to $n$; $t$ is the first year of life lost, and $n$ is the final year of the total number of years of life lost per EVD death within an age group, which is obtained by subtracting the age groups average age at death (GAAD) for EVD-related causes from the DRC average life expectancy at birth; $NHGPC_{ Int\$}$is per capita non-health GDP in purchasing power parity (PPP), which is obtained by subtracting per capita current health expenditure ($PCCHE$) from GDP per capita ($GDPPC_{ Int\$}$); $EVDD_{1-4}$ is the EVD deaths between the age of 1–4 years; $EVDD_{5-9}$ is the EVD deaths between the age of 5–9 years; $EVDD_{10-14}$ is the EVD deaths between the age of 10–14 years; $EVDD_{15-19}$ is the EVD deaths between the age of 15–19 years; $EVDD_{20-24}$ is the EVD deaths between the age of 20–24 years; $EVDD_{25-29}$ is the EVD deaths between the age of 25–29 years; $EVDD_{30-34}$ is the EVD deaths between the age of 30–34 years; $EVDD_{35-39}$ is the EVD deaths between the age of 35–39 years; $EVDD_{40-44}$ is the EVD deaths between the age of 40–44 years; $EVDD_{45-49}$ is the EVD deaths between the age of 45–49 years; $EVDD_{50-54}$ is the EVD deaths between the age of 54–54 years; $EVDD_{55-59}$ is the EVD deaths between the age of 55–59 years; $EVDD_{60-64}$ is the EVD deaths between the age of 60–64 years; $EVDD_{65-69}$ is the EVD deaths between the age of 65–69 years; $EVDD_{70-74}$ is the EVD deaths between the age of 70–74 years; $EVDD_{75-79}$ is the EVD deaths between the age of 70–74 years; $EVDD_{75-79}$ is the EVD deaths between the age of 75–79 years; $EVDD_{80-84}$ is the EVD deaths between the age of 80–84 years; $EVDD_{85-89}$ is the EVD deaths between the age of 85–89 years; $EVDD_{90-94}$ is the EVD deaths between the age of 90–94 years; and $EVDD_{\geq95}$ is the EVD deaths between the age of 95 years and above in DRC.
