# Supplementary material for: The monetary value of human lives lost through Ebola virus disease in the Democratic Republic of Congo in 2019
Source: BMC Public Health. 2019 Sep 3;19:1218. doi: 10.1186/s12889-019-7542-2 (PMC6724278; doi:10.1186/s12889-019-7542-2)
Supplement: Supplementary file 3 — Age distribution of EVD deaths in DRC. (DOCX 12 kb) [file 12889_2019_7542_MOESM3_ESM.docx]

| **Additional File 3: Age distribution of EVD deaths in DRC** | | |
| --- | --- | --- |
| Age Group | (A). Proportions^1^ | (B). Deaths by age group [B=A x 1286 EVD deaths]^2^ |
| 1 to 4 | 0.22857 | 294 |
| 5 to 9 | 0.097403 | 125 |
| 10 to 14 | 0.048936 | 63 |
| 15 to 19 | 0.034719 | 45 |
| 20 to 24 | 0.029532 | 38 |
| 25 to 29 | 0.027115 | 35 |
| 30 to 34 | 0.027054 | 35 |
| 35 to 39 | 0.300416 | 387 |
| 40 to 44 | 0.029707 | 38 |
| 45 to 49 | 0.031268 | 40 |
| 50 to 54 | 0.032936 | 42 |
| 55 to 59 | 0.031355 | 40 |
| 60 to 64 | 0.027201 | 35 |
| 65 to 69 | 0.020045 | 26 |
| 70 to 74 | 0.014579 | 19 |
| 75 to 79 | 0.010056 | 13 |
| 80 to 84 | 0.00579 | 7 |
| 85 to 89 | 0.002495 | 3 |
| 90 to 94 | 0.000678 | 1 |
| 95 plus | 0.000145 | 0 |

Source: ^1^IHME [27]. ^2^DRC Ministry of Health [5].
