# Supplementary material for: The monetary value of human lives lost through Ebola virus disease in the Democratic Republic of Congo in 2019
Source: BMC Public Health. 2019 Sep 3;19:1218. doi: 10.1186/s12889-019-7542-2 (PMC6724278; doi:10.1186/s12889-019-7542-2)
Supplement: Supplementary file 4 — Undiscounted potential years of life lost from EVD assuming DRC life expectancy. (DOCX 12 kb) [file 12889_2019_7542_MOESM4_ESM.docx]

| **Additional File 4: Undiscounted potential years of life lost from EVD assuming DRC life expectancy** | | | |
| --- | --- | --- | --- |
| **Age Group** | **(A). Group average age at death** | **(B). Years of Life Lost [C=60.5years - A)]** | **(C). Potentially Productive Years of Life Lost** |
| 1 – 4 | 2.5 | 58 | 46.5 |
| 5 – 9 | 7 | 53.5 | 46.5 |
| 10 – 14 | 12 | 48.5 | 46.5 |
| 15 – 19 | 17 | 43.5 | 43.5 |
| 20 – 24 | 22 | 38.5 | 38.5 |
| 25 – 29 | 27 | 33.5 | 33.5 |
| 30 – 34 | 32 | 28.5 | 28.5 |
| 35 – 39 | 37 | 23.5 | 23.5 |
| 40 – 44 | 42 | 18.5 | 18.5 |
| 45 – 49 | 47 | 13.5 | 13.5 |
| 50 – 54 | 52 | 8.5 | 8.5 |
| 55 – 59 | 57 | 3.5 | 3.5 |
| 60 – 64^*^ | 62 | 0 | 0 |
| 65 – 69^*^ | 67 | 0 | 0 |
| 70 – 74^*^ | 72 | 0 | 0 |
| 75 – 79^*^ | 77 | 0 | 0 |
| 80 – 84* | 82 | 0 | 0 |
| 85 – 89^*^ | 87 | 0 | 0 |
| 90 – 94^*^ | 92 | 0 | 0 |
| =>95^*^ | 95 | 0 | 0 |

Source: Authors calculations. Note: ^*^PYLL cannot be negative. Thus, for age groups with average age at death greater than DRC life average life expectancy of 60.5 years, their PYLL are assumed to be equal to zero [31].
