# Supplementary material for: The monetary value of human lives lost through Ebola virus disease in the Democratic Republic of Congo in 2019
Source: BMC Public Health. 2019 Sep 3;19:1218. doi: 10.1186/s12889-019-7542-2 (PMC6724278; doi:10.1186/s12889-019-7542-2)
Supplement: Supplementary file 5 — Undiscounted potential years of life lost from EVD assuming world’s and Japanese Female life expectancies. (DOCX 13 kb) [file 12889_2019_7542_MOESM5_ESM.docx]

**Additional File 5: Undiscounted potential years of life lost from EVD assuming world’s and Japanese Female life expectancies**

| **A: Undiscounted potential years of life lost from EVD assuming world’s life expectancy** | | | |
| --- | --- | --- | --- |
| Age Group | (A). Group average age at death | (B). Years of Life Lost [C=72 years - A)] | (C). Potentially Productive Years of Life Lost |
| 1 – 4 | 2.5 | 69.5 | 58 |
| 5 – 9 | 7 | 65 | 58 |
| 10 – 14 | 12 | 60 | 58 |
| 15 – 19 | 17 | 55 | 55 |
| 20 – 24 | 22 | 50 | 50 |
| 25 – 29 | 27 | 45 | 45 |
| 30 – 34 | 32 | 40 | 40 |
| 35 – 39 | 37 | 35 | 35 |
| 40 – 44 | 42 | 30 | 30 |
| 45 – 49 | 47 | 25 | 25 |
| 50 – 54 | 52 | 20 | 20 |
| 55 – 59 | 57 | 15 | 15 |
| 60 – 64 | 62 | 10 | 10 |
| 65 – 69 | 67 | 5 | 5 |
| 70 – 74^*^ | 72 | 0 | 0 |
| 75 – 79^*^ | 77 | 0 | 0 |
| 80 – 84* | 82 | 0 | 0 |
| 85 – 89^*^ | 87 | 0 | 0 |
| 90 – 94^*^ | 92 | 0 | 0 |
| =>95^*^ | 95 | 0 | 0 |

Source: Authors calculations using the world average life expectancy from WHO [6]. Note: ^*^PYLL cannot be negative. Thus, for age groups with average age at death greater than DRC life average life expectancy of 72 years, their PYLL are assumed to be equal to zero [31].

| **B: Undiscounted potential years of life lost from EVD assuming the Japan female life expectancy** | | | |
| --- | --- | --- | --- |
| Age Group | (A). Group average age at death | (B). Years of Life Lost [C=87 years – (A)] | (C). Potentially Productive Years of Life Lost |
| 1 – 4 | 2.5 | 84.5 | 73 |
| 5 – 9 | 7 | 80 | 73 |
| 10 – 14 | 12 | 75 | 73 |
| 15 – 19 | 17 | 70 | 70 |
| 20 – 24 | 22 | 65 | 65 |
| 25 – 29 | 27 | 60 | 60 |
| 30 – 34 | 32 | 55 | 55 |
| 35 – 39 | 37 | 50 | 50 |
| 40 – 44 | 42 | 45 | 45 |
| 45 – 49 | 47 | 40 | 40 |
| 50 – 54 | 52 | 35 | 35 |
| 55 – 59 | 57 | 30 | 30 |
| 60 – 64 | 62 | 25 | 25 |
| 65 – 69 | 67 | 20 | 20 |
| 70 – 74 | 72 | 15 | 15 |
| 75 – 79 | 77 | 10 | 10 |
| 80 – 84 | 82 | 5 | 5 |
| 85 – 89 | 87 | 0 | 0 |
| 90 – 94 | 92 | 0 | 0 |
| =>95 | 95 | 0 | 0 |

Source: Authors calculations using Japan female life expectancy from WHO [6]. Note: ^*^PYLL cannot be negative. Thus, for age groups with average age at death greater than DRC life average life expectancy of 87 years, their PYLL are assumed to be equal to zero [31].
