# Supplementary material for: The monetary value of human lives lost through Ebola virus disease in the Democratic Republic of Congo in 2019
Source: BMC Public Health. 2019 Sep 3;19:1218. doi: 10.1186/s12889-019-7542-2 (PMC6724278; doi:10.1186/s12889-019-7542-2)
Supplement: Supplementary file 6 — Discounted potential years of life lost from EVD assuming DRC life expectancy and 3% discount rate. (DOCX 12 kb) [file 12889_2019_7542_MOESM6_ESM.docx]

| **Additional File 6: Discounted potential years of life lost from EVD assuming DRC life expectancy and 3% discount rate** | | |
| --- | --- | --- |
| **Age Group** | **Potentially Productive Years of Life Lost** | **Productive Years of Life Lost discounted at 3%** |
| 1 – 4 | 46.5 | 25.02470783 |
| 5 – 9 | 46.5 | 25.02470783 |
| 10 – 14 | 46.5 | 25.02470783 |
| 15 – 19 | 43.5 | 24.25427392 |
| 20 – 24 | 38.5 | 22.80821513 |
| 25 – 29 | 33.5 | 21.13183668 |
| 30 – 34 | 28.5 | 19.18845459 |
| 35 – 39 | 23.5 | 16.93554212 |
| 40 – 44 | 18.5 | 14.32379911 |
| 45 – 49 | 13.5 | 11.29607314 |
| 50 – 54 | 8.5 | 7.786108922 |
| 55 – 59 | 3.5 | 3.717098403 |
| 60 – 64 | 0 | 0 |
| 65 – 69 | 0 | 0 |
| 70 – 74 | 0 | 0 |
| 75 – 79 | 0 | 0 |
| 80 – 84 | 0 | 0 |
| 85 – 89 | 0 | 0 |
| 90 – 94 | 0 | 0 |
| =>95 | 0 | 0 |

Source: Author calculations.
