# Supplementary material for: The monetary value of human lives lost through Ebola virus disease in the Democratic Republic of Congo in 2019
Source: BMC Public Health. 2019 Sep 3;19:1218. doi: 10.1186/s12889-019-7542-2 (PMC6724278; doi:10.1186/s12889-019-7542-2)
Supplement: Supplementary file 7 — Discounted potential years of life lost from EVD assuming world’s and Japan Female life expectancies and a 3% discount rate. (DOCX 13 kb) [file 12889_2019_7542_MOESM7_ESM.docx]

**Additional File 7: Discounted potential years of life lost from EVD assuming world’s and Japan Female life expectancies and a 3% discount rate**

| A: Discounted potential years of life lost from EVD assuming world’s life expectancy (3% discount rate) | | |
| --- | --- | --- |
| Age Group | Potentially Productive Years of Life Lost | Productive Years of Life Lost discounted at 3% |
| 1 – 4 | 58 | 27.33100549 |
| 5 – 9 | 58 | 27.33100549 |
| 10 – 14 | 58 | 27.33100549 |
| 15 – 19 | 55 | 26.77442764 |
| 20 – 24 | 50 | 25.72976401 |
| 25 – 29 | 45 | 24.51871254 |
| 30 – 34 | 40 | 23.11477197 |
| 35 – 39 | 35 | 21.48722007 |
| 40 – 44 | 30 | 19.60044135 |
| 45 – 49 | 25 | 17.41314769 |
| 50 – 54 | 20 | 14.87747486 |
| 55 – 59 | 15 | 11.93793509 |
| 60 – 64 | 10 | 8.530202837 |
| 65 – 69 | 5 | 4.579707187 |
| 70 – 74 | 0 | 0 |
| 75 – 79 | 0 | 0 |
| 80 – 84 | 0 | 0 |
| 85 – 89 | 0 | 0 |
| 90 – 94 | 0 | 0 |
| =>95 | 0 | 0 |

Source: Authors calculations.

| Table B: Discounted potential years of life lost from EVD assuming the Japan female life expectancy (3% discount rate) | | |
| --- | --- | --- |
| Age Group | Potentially Productive Years of Life Lost | Productive Years of Life Lost discounted at 3% |
| 1 – 4 | 73 | 29.4806675 |
| 5 – 9 | 73 | 29.4806675 |
| 10 – 14 | 73 | 29.4806675 |
| 15 – 19 | 70 | 29.12342135 |
| 20 – 24 | 65 | 28.45289152 |
| 25 – 29 | 60 | 27.67556367 |
| 30 – 34 | 55 | 26.77442764 |
| 35 – 39 | 50 | 25.72976401 |
| 40 – 44 | 45 | 24.51871254 |
| 45 – 49 | 40 | 23.11477197 |
| 50 – 54 | 35 | 21.48722007 |
| 55 – 59 | 30 | 19.60044135 |
| 60 – 64 | 25 | 17.41514769 |
| 65 – 69 | 20 | 14.87747486 |
| 70 – 74 | 15 | 11.93793509 |
| 75 – 79 | 10 | 8.530202837 |
| 80 – 84 | 5 | 4.579707187 |
| 85 – 89 | 0 | 0 |
| 90 – 94 | 0 | 0 |
| =>95 | 0 | 0 |

Source: Authors calculations.
