# Supplementary material for: The monetary value of human lives lost through Ebola virus disease in the Democratic Republic of Congo in 2019
Source: BMC Public Health. 2019 Sep 3;19:1218. doi: 10.1186/s12889-019-7542-2 (PMC6724278; doi:10.1186/s12889-019-7542-2)
Supplement: Supplementary file 8 — Discounted potential years of life lost from EVD assuming the DRC, the world and Japanese female life expectancies and a 5% discount rate. (DOCX 13 kb) [file 12889_2019_7542_MOESM8_ESM.docx]

**Additional File 8: Discounted potential years of life lost from EVD assuming the DRC, the world and Japanese female life expectancies and a 5% discount rate**

| **A: Discounted potential years of life lost from EVD assuming DRC life expectancy (5% discount rate)** | | |
| --- | --- | --- |
| Age Group | Potentially Productive Years of Life Lost | Productive Years of Life Lost discounted at 5% |
| 1 – 4 | 46.5 | 17.98101571 |
| 5 – 9 | 46.5 | 17.98101571 |
| 10 – 14 | 46.5 | 17.98101571 |
| 15 – 19 | 43.5 | 17.66277331 |
| 20 – 24 | 38.5 | 17.01704067 |
| 25 – 29 | 33.5 | 16.19290401 |
| 30 – 34 | 28.5 | 15.14107358 |
| 35 – 39 | 23.5 | 13.79864179 |
| 40 – 44 | 18.5 | 12.08532086 |
| 45 – 49 | 13.5 | 9.89864094 |
| 50 – 54 | 8.5 | 7.107821676 |
| 55 – 59 | 3.5 | 3.545950504 |
| 60 – 64 | 0 | 0 |
| 65 – 69 | 0 | 0 |
| 70 – 74 | 0 | 0 |
| 75 – 79 | 0 | 0 |
| 80 – 84 | 0 | 0 |
| 85 – 89 | 0 | 0 |
| 90 – 94 | 0 | 0 |
| =>95 | 0 | 0 |

Source: Author calculations.

| **B: Discounted potential years of life lost from EVD assuming world’s life expectancy (5% discount rate)** | | |
| --- | --- | --- |
| Age Group | Potentially Productive Years of Life Lost | Productive Years of Life Lost discounted at 5% |
| 1 – 4 | 58 | 18.8195417 |
| 5 – 9 | 58 | 18.8195417 |
| 10 – 14 | 58 | 18.8195417 |
| 15 – 19 | 55 | 18.63347196 |
| 20 – 24 | 50 | 18.25592546 |
| 25 – 29 | 45 | 17.77406982 |
| 30 – 34 | 40 | 17.15908635 |
| 35 – 39 | 35 | 16.37419429 |
| 40 – 44 | 30 | 15.37245103 |
| 45 – 49 | 25 | 14.09394457 |
| 50 – 54 | 20 | 12.46221034 |
| 55 – 59 | 15 | 10.37965804 |
| 60 – 64 | 10 | 7.721734929 |
| 65 – 69 | 5 | 4.329476671 |
| 70 – 74 | 0 | 0 |
| 75 – 79 | 0 | 0 |
| 80 – 84 | 0 | 0 |
| 85 – 89 | 0 | 0 |
| 90 – 94 | 0 | 0 |
| =>95 | 0 | 0 |

Source: Authors calculations.

| C: Discounted potential years of life lost from EVD assuming the Japan female life expectancy (5% discount rate) | | |
| --- | --- | --- |
| Age Group | Potentially Productive Years of Life Lost | Productive Years of Life Lost discounted at 5% |
| 1 – 4 | 73 | 19.43217937 |
| 5 – 9 | 73 | 19.43217937 |
| 10 – 14 | 73 | 19.43217937 |
| 15 – 19 | 70 | 19.34267667 |
| 20 – 24 | 65 | 19.16107033 |
| 25 – 29 | 60 | 18.92928953 |
| 30 – 34 | 55 | 18.63347196 |
| 35 – 39 | 50 | 18.25592546 |
| 40 – 44 | 45 | 17.77406982 |
| 45 – 49 | 40 | 17.15908635 |
| 50 – 54 | 35 | 16.37419429 |
| 55 – 59 | 30 | 15.37245103 |
| 60 – 64 | 25 | 14.09394457 |
| 65 – 69 | 20 | 12.46221034 |
| 70 – 74 | 15 | 10.37965804 |
| 75 – 79 | 10 | 7.721734929 |
| 80 – 84 | 5 | 4.329476671 |
| 85 – 89 | 0 | 0 |
| 90 – 94 | 0 | 0 |
| =>95^*^ | 0 | 0 |

Source: Authors calculations.
