# Supplementary material for: The monetary value of human lives lost through Ebola virus disease in the Democratic Republic of Congo in 2019
Source: BMC Public Health. 2019 Sep 3;19:1218. doi: 10.1186/s12889-019-7542-2 (PMC6724278; doi:10.1186/s12889-019-7542-2)
Supplement: Supplementary file 9 — Discounted potential years of life lost from EVD assuming the DRC, the World and the Japan female life expectancies and a 10% discount rate. (DOCX 13 kb) [file 12889_2019_7542_MOESM9_ESM.docx]

**Additional File 9: Discounted potential years of life lost from EVD assuming the DRC, the World and the Japan female life expectancies and a 10% discount rate**

| A: Discounted potential years of life lost from EVD assuming DRC life expectancy (10% discount rate) | | |
| --- | --- | --- |
| Age Group | Potentially Productive Years of Life Lost | Productive Years of Life Lost discounted at 10% |
| 1 – 4 | 46.5 | 9.886618082 |
| 5 – 9 | 46.5 | 9.886618082 |
| 10 – 14 | 46.5 | 9.886618082 |
| 15 – 19 | 43.5 | 9.849088668 |
| 20 – 24 | 38.5 | 9.75695579 |
| 25 – 29 | 33.5 | 9.60857487 |
| 30 – 34 | 28.5 | 9.369605914 |
| 35 – 39 | 23.5 | 8.98474402 |
| 40 – 44 | 18.5 | 8.364920092 |
| 45 – 49 | 13.5 | 7.366687457 |
| 50 – 54 | 8.5 | 5.759023816 |
| 55 – 59 | 3.5 | 3.169865446 |
| 60 – 64 | 0 | 0 |
| 65 – 69 | 0 | 0 |
| 70 – 74 | 0 | 0 |
| 75 – 79 | 0 | 0 |
| 80 – 84 | 0 | 0 |
| 85 – 89 | 0 | 0 |
| 90 – 94 | 0 | 0 |
| =>95 | 0 | 0 |

Source: Author calculations.

| B: Discounted potential years of life lost from EVD assuming world’s life expectancy (10% discount rate) | | |
| --- | --- | --- |
| Age Group | Potentially Productive Years of Life Lost | Productive Years of Life Lost discounted at 10% |
| 1 – 4 | 58 | 9.96026033 |
| 5 – 9 | 58 | 9.96026033 |
| 10 – 14 | 58 | 9.96026033 |
| 15 – 19 | 55 | 9.947106499 |
| 20 – 24 | 50 | 9.914814487 |
| 25 – 29 | 45 | 9.86280788 |
| 30 – 34 | 40 | 9.779050718 |
| 35 – 39 | 35 | 9.644158973 |
| 40 – 44 | 30 | 9.426914467 |
| 45 – 49 | 25 | 9.077040018 |
| 50 – 54 | 20 | 8.51356372 |
| 55 – 59 | 15 | 7.606079506 |
| 60 – 64 | 10 | 6.144567106 |
| 65 – 69 | 5 | 3.790786769 |
| 70 – 74 | 0 | 0 |
| 75 – 79 | 0 | 0 |
| 80 – 84 | 0 | 0 |
| 85 – 89 | 0 | 0 |
| 90 – 94 | 0 | 0 |
| =>95 | 0 | 0 |

Source: Authors calculations.

| C: Discounted potential years of life lost from EVD assuming the Japan female life expectancy (10% discount rate) | | |
| --- | --- | --- |
| Age Group | Potentially Productive Years of Life Lost | Productive Years of Life Lost discounted at 10% |
| 1 – 4 | 73 | 9.990486639 |
| 5 – 9 | 73 | 9.990486639 |
| 10 – 14 | 73 | 9.990486639 |
| 15 – 19 | 70 | 9.987337716 |
| 20 – 24 | 65 | 9.979607266 |
| 25 – 29 | 60 | 9.967157297 |
| 30 – 34 | 55 | 9.947106499 |
| 35 – 39 | 50 | 9.914814487 |
| 40 – 44 | 45 | 9.86280788 |
| 45 – 49 | 40 | 9.779050718 |
| 50 – 54 | 35 | 9.644158973 |
| 55 – 59 | 30 | 9.426914467 |
| 60 – 64 | 25 | 9.077040018 |
| 65 – 69 | 20 | 8.51356372 |
| 70 – 74 | 15 | 7.606079506 |
| 75 – 79 | 10 | 6.144567106 |
| 80 – 84 | 5 | 3.790786769 |
| 85 – 89 | 0 | 0 |
| 90 – 94 | 0 | 0 |
| =>95 | 0 | 0 |

Source: Authors calculations.
